# Housekeeping Gene Selection Advisory: Glyceraldehyde-3-Phosphate Dehydrogenase (GAPDH) and β-Actin Are Targets of miR-644a

**DOI:** 10.1371/journal.pone.0047510

**Published:** 2012-10-16

**Authors:** Kavleen Sikand, Jagjit Singh, Jey Sabith Ebron, Girish C. Shukla

**Affiliations:** 1 Department of Biological, Geological and Environmental Sciences, Cleveland State University, Cleveland, Ohio, United States of America; 2 Center for Gene Regulation in Health and Disease, Cleveland State University, Cleveland, Ohio, United States of America; German Cancer Research Center, Germany

## Abstract

Results of overexpression or downregulation of a microRNA (miRNA) on its target mRNA expression are often validated by reverse-transcription and quantitative PCR analysis using an appropriate housekeeping gene as an internal control. The possible direct or indirect effects of a miRNA on the expression of housekeeping genes are often overlooked. Among many housekeeping genes, expressions of glyceraldehyde-3-phosphate dehydrogenase (GAPDH) and β-actin have been used extensively for normalization of gene expression data. Here, we show that GAPDH and β-actin are direct targets of miR-644a. Our data demonstrate the unsuitability of GAPDH and β-actin as internal controls in miR-644a functional studies and emphasize the need to carefully consider the choice of a reference gene in miRNA experiments.

## Introduction

The category of housekeeping genes consists of genes that are involved in the regulation of basic and ubiquitous cellular functions required for the survival of most cell types [1]. Due to their presumptive invariable expression, housekeeping genes have been used extensively as reference genes for normalization of gene expression data derived from a variety of cell types or experimental treatments using microarray or quantitative reverse-transcriptase polymerase chain reaction (qRT-PCR). A reference gene is necessary to correct for basic sample differences, including differences in cellular input, RNA quality, efficiency of reverse transcription and batch to batch variation in reagents. Some of the housekeeping genes commonly used as expression controls include glyceraldehyde-3-phosphate dehydrogenase (GAPDH), β-actin, β_2_-microglobulin, cyclooxygenase 1, hypoxanthine phosphoribosyl transferase 1, glucose-6-phosphate dehydrogenase, cyclophilin A, tubulin, transferrin receptor and 18S ribosomal RNA [2], [3].

Changes in mRNA expression profile in response to microRNA (miRNA) inhibition and/or overexpression provide important information about the function of a miRNA. The housekeeping genes, GAPDH and β-actin are routinely used for the normalization of data in qRT-PCR experiments estimating the effect of miRNAs on target mRNA expression. These studies assume unaltered expression of GAPDH and β-actin in the presence of ectopically overexpressed miRNAs without giving due consideration to the possibility of miRNA-mediated effects on the expression of these genes. A single miRNA has the potential to target the expression of multiple genes. These target genes may include the housekeeping gene being considered for internal control, thus making it an inappropriate control gene in settings where the miRNA is the experimental treatment given to cells. Here, we show that miR-644a significantly represses GAPDH and β-actin expression. We also show that miR-644a functions by directly binding to its target site in the 3′ untranslated region (UTR) of GAPDH and β-actin. Our results reinforce the earlier view of using caution and proper validation while selecting reference genes for a particular experimental setting.

## Results and Discussion

### miR-644a Downregulates GAPDH and β-actin Expression

**Figure 1:**
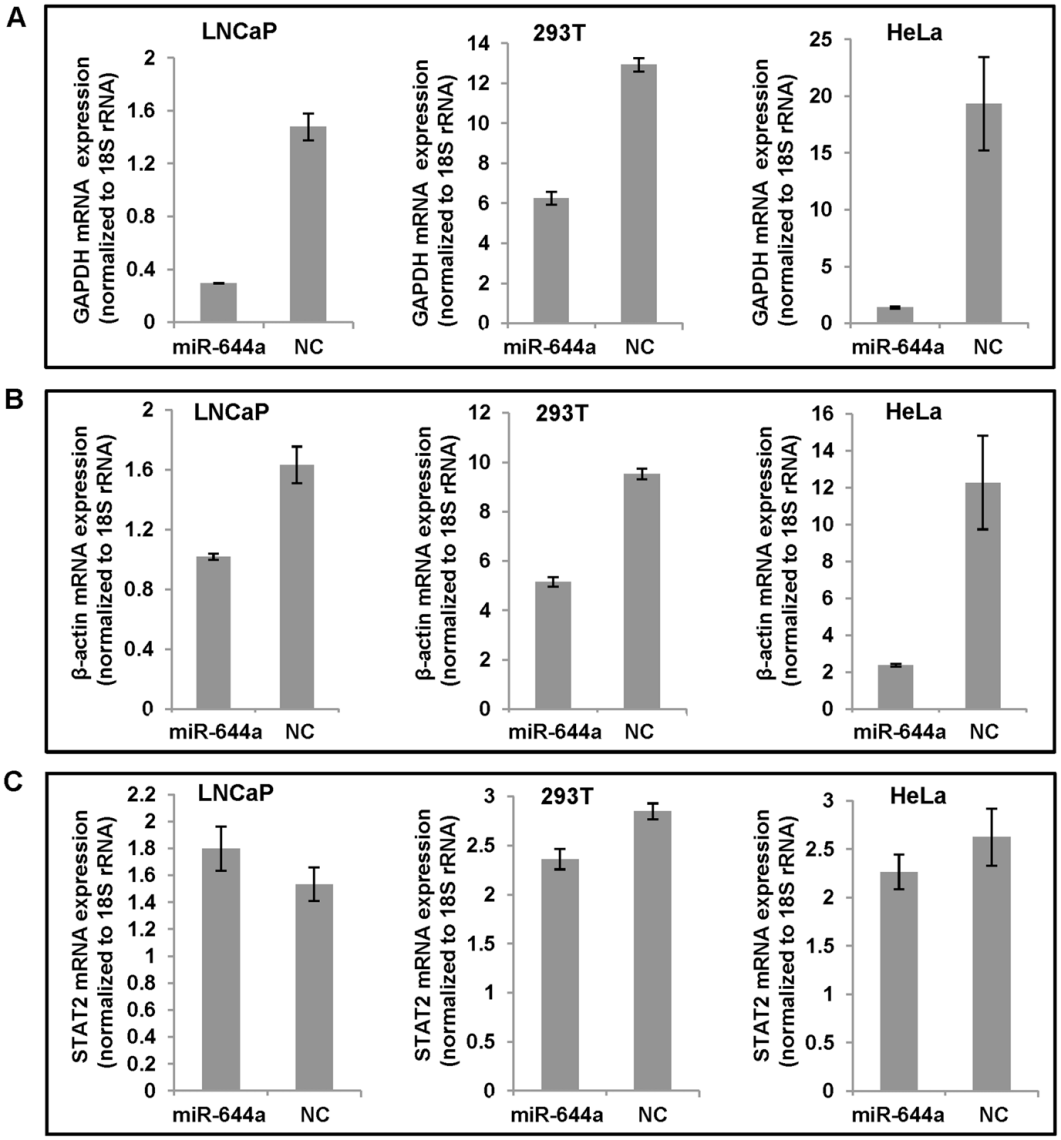
miR-644a downregulates GAPDH and β-actin mRNA expression. (A and B) Quantitative real-time PCR analysis of GAPDH and β-actin mRNA expression in LNCaP, 293T and HeLa cells transfected with miR-644a mimic or negative control (NC) mimic. (C) In order to demonstrate that the repression of GAPDH and β-actin mRNA expression is a consequence of specific targeting by miR-644a, the effect of miR-644a was checked on a computationally predicted non-target gene, STAT2. STAT2 mRNA expression was determined by quantitative real-time PCR analysis in LNCaP, 293T and HeLa cells transfected with miR-644a mimic or NC mimic. GAPDH, β-actin and STAT2 mRNA expression was normalized to 18S rRNA expression. Data are plotted as mean ± SE of three independent experiments.

**Figure 2:**
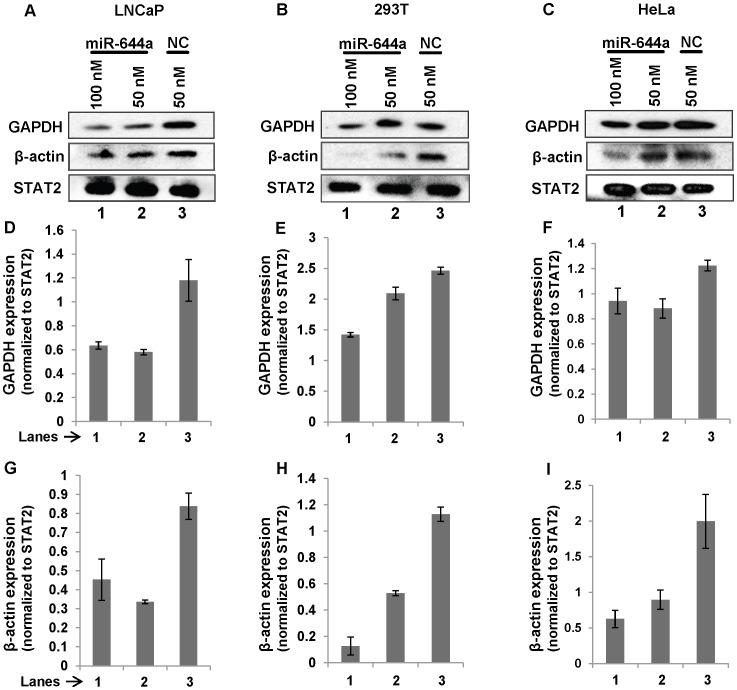
miR-644a downregulates GAPDH and β-actin protein expression. (A, B and C) Representative western blots showing the expression of GAPDH, β-actin and STAT2 in LNCaP, 293T and HeLa cells treated with indicated amounts of miR-644a mimic or negative control (NC) mimic for 48 hours. STAT2 expression was used as a loading control. (D, E and F) Quantitation of GAPDH protein expression in the respective lanes as shown in A, B and C. (G, H and I) Quantitation of β-actin protein expression in the respective lanes as shown in A, B and C. Three independent western blots were used for the quantification of protein expression. The signal intensities of bands were measured using ImageJ software. The GAPDH or β-actin expression in each lane was determined by normalizing GAPDH or β-actin band intensity to STAT2 band intensity. Data are plotted as mean ± SE of three independent experiments.

While studying the effect of a panel of miRNAs on target mRNA expression in prostate cancer cell lines, we found that the use of GAPDH or β-actin as normalization control in case of miR-644a treatment yielded misleading results causing us to suspect the repression of GAPDH and β-actin expression by miR-644a. To confirm this, we evaluated the effect of miR-644a on GAPDH and β-actin expression in a panel of three cell lines: LNCaP (human prostate cancer), HEK 293T (human embryonic kidney) and HeLa (human cervical cancer). Cells were transfected with miR-644a mimic or negative control (NC) mimic. GAPDH and β-actin mRNA/protein levels were measured 48 hours post-transfection. 18S ribosomal RNA (rRNA) expression was used as an internal control for normalization of GAPDH and β-actin mRNA expression. GAPDH and β-actin protein levels were normalized to signal transducer and activator of transcription 2 (STAT2) expression. Our computational analysis confirmed that STAT2 open reading frame including 5′ and 3′ UTRs does not appear to contain any miR-644a target site that follows established miRNA-target mRNA base-pairing rules. Nevertheless, we investigated if STAT2 mRNA expression is affected by miR-644a transfection. As seen in figure 1A, miR-644a reduced GAPDH mRNA levels by 50% to 90% as compared to NC mimic in LNCaP, 293T and HeLa cells. Similar reduction was observed in β-actin mRNA expression in the three cell lines transfected with miR-644a mimic (Figure 1B). As expected, miR-644a failed to inhibit the expression of STAT2 mRNA (Figure 1C). These data confirmed that GAPDH and β-actin are targets of miR-644a and STAT2 is not. Furthermore, western blots showed the repression of GAPDH and β-actin protein levels by miR-644a transfection in LNCaP, 293T and HeLa cells (Figure 2). Taken together, these data provide evidence for miR-644a-mediated regulation of GAPDH and β-actin expression and hence, indicate the unsuitability of these housekeeping genes as internal controls in experiments involving miR-644a. A recent study [4] designed to evaluate the androgen receptor-targeting miRNAs has also noted the repressive effect of miR-644a on GAPDH mRNA expression; however, this study did not report any repressive effect of miR-644a on β-actin mRNA expression, which was used as an endogenous control instead of GAPDH. Also, no further attempts were made to evaluate if GAPDH is a direct target of miR-644a.

### miR-644a Directly Targets GAPDH and β-actin 3′ UTRs

**Figure 3:**
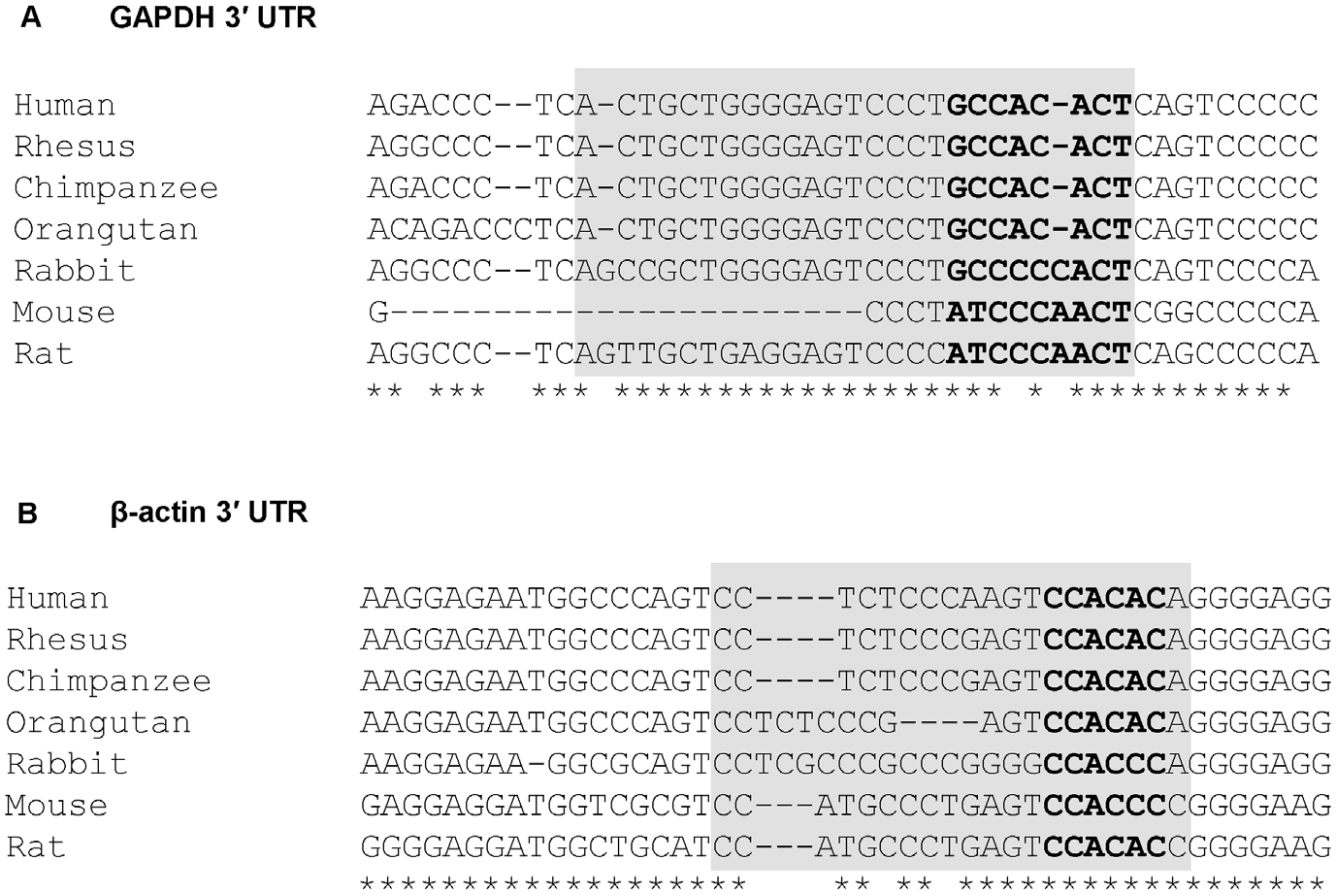
Conservation of miR-644a target site. Panels A and B show alignments of GAPDH and β-actin 3′ UTR sequences containing miR-644a binding site in 7 mammalian species. miR-644a target site sequence is shown in gray box and seed binding region is shown in bold. Stars indicate conserved nucleotides in the target sequence in at least 5 out of 7 species.

**Figure 4:**
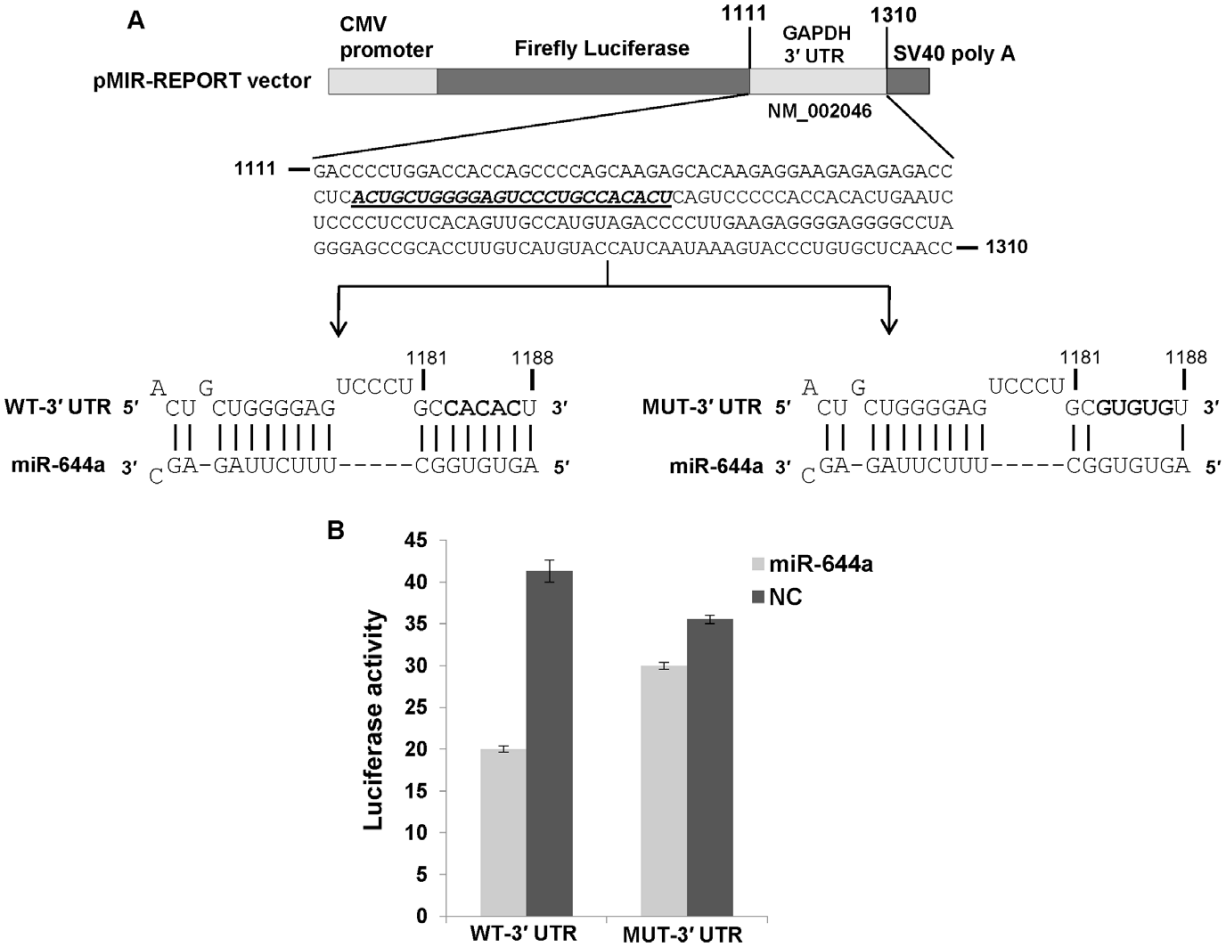
GAPDH is a direct target of miR-644a. (A) Schematic representation of firefly luciferase reporter construct containing GAPDH 3′ UTR with either wild type (WT) or mutant (MUT) miR-644a target site. The miR-644a target site in GAPDH 3′ UTR is italicized and underlined. In the MUT-3′ UTR construct, 5 nucleotides (1183–1187) in the seed binding region of the target site were mutated to their complementary nucleotides (shown in bold) in order to disrupt miR-644a binding. (B) Luciferase reporter assay in CHO-K1 cells cotransfected with WT-3′ UTR or MUT-3′ UTR construct and miR-644a mimic (2 nM) or negative control (NC) mimic (2 nM) as indicated. Renilla luciferase reporter plasmid was cotransfected in all cases as a control for transfection efficiency. Luciferase activity is plotted as a ratio of firefly to renilla luciferase activity. Each bar represents mean ± SE of three independent experiments.

**Figure 5:**
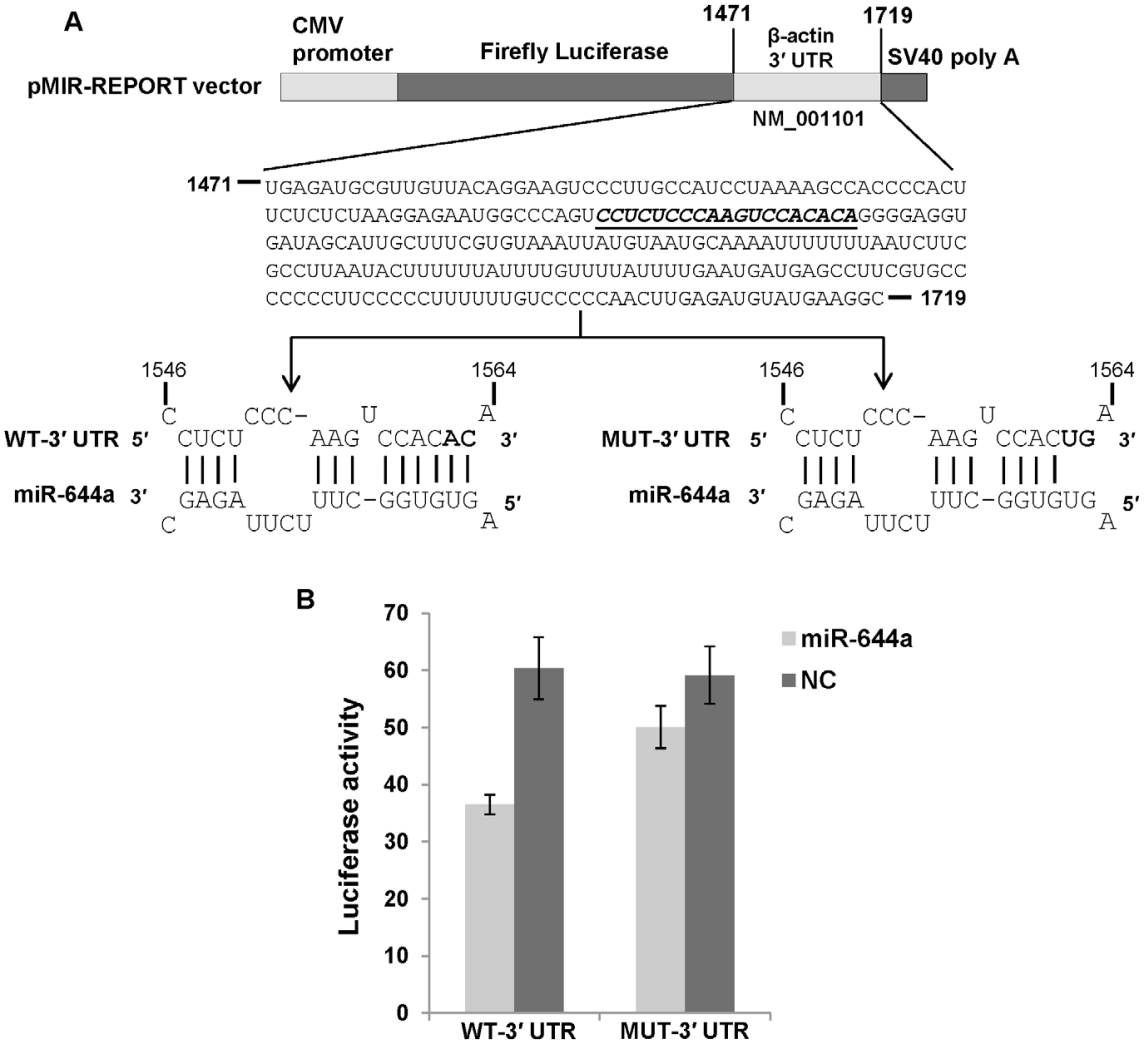
β-actin is a direct target of miR-644a. (A) Schematic representation of firefly luciferase reporter construct containing β-actin 3′ UTR with either wild type (WT) or mutant (MUT) miR-644a target site. The italicized and underlined sequence in β-actin 3′ UTR represents the miR-644a target site. In the MUT-3′ UTR construct, 2 nucleotides (1562–1563) in the seed binding region of the target site were mutated to their complementary nucleotides (shown in bold) in order to disrupt miR-644a binding. (B) Luciferase reporter assay in CHO-K1 cells cotransfected with WT-3′ UTR or MUT-3′ UTR construct and miR-644a mimic (2 nM) or negative control (NC) mimic (2 nM) as indicated. Renilla luciferase reporter plasmid was cotransfected in all cases as a control for transfection efficiency. Luciferase activity is plotted as a ratio of firefly to renilla luciferase activity. Each bar represents mean ± SE of three independent experiments.

Next, we asked if the observed reduction in GAPDH and β-actin mRNA levels is a consequence of miR-644a interacting with the 3′ UTRs of these mRNAs. A search for GAPDH- and β-actin-targeting miRNAs using TargetScan Human (release 6.1) revealed a potential binding site for miR-644a in GAPDH 3′ UTR and β-actin 3′ UTR suggesting that miR-644a could be directly regulating the expression of these genes by binding to the predicted target sites. We checked the evolutionary conservation of miR-644a target site in GAPDH 3′ UTR (Figure 3A) and β-actin 3′ UTR (Figure 3B) in seven mammalian genomes. The seed binding region of miR-644a target site (shown in bold, Figures 3A, B) was found to be highly conserved in both sets of 3′ UTRs. In order to validate the direct interaction of miR-644a with its cognate target site, we cloned GAPDH 3′ UTR containing the wild type (WT) or mutated (MUT) miR-644a target site in a firefly luciferase reporter vector (Figure 4A). Similar luciferase reporter constructs were made using a segment of β-actin 3′ UTR (Figure 5A). In the GAPDH MUT-3′ UTR construct, nucleotides 1183 to 1187 of the target site were mutated to their complementary nucleotides to disrupt any potential base-pairing interaction of miR-644a (Figure 4A). In the β-actin MUT-3′ UTR construct, nucleotides 1562 and 1563 of miR-644a binding site were mutated (Figure 5A). Each reporter construct (WT-3′ UTR or MUT-3′ UTR) was cotransfected with either miR-644a mimic or NC mimic in CHO-K1 cells and luciferase activity was measured 24 hours post-transfection. In experiments where miR-644a mimic was cotransfected with WT-3′ UTR luciferase reporter construct, we observed a marked repression of luciferase activity (Figures 4B and 5B). As expected, in experiments where miR-644a mimic was cotransfected with MUT-3′ UTR construct, a reversal of luciferase expression was observed (Figures 4B and 5B). Taken together, these data show that miR-644a represses GAPDH and β-actin expression by directly interacting with its target sequence in the respective 3′ UTRs.

Our results show robust reduction in GAPDH and β-actin mRNA expression and luciferase reporter activity by overexpressed miR-644a. However, whether endogenous miR-644a plays a significant role in the modulation of GAPDH and β-actin expression needs further investigation. Interestingly, we could not detect the endogenous expression of miR-644a in several cancer cell lines, including LNCaP and HeLa using TaqMan MicroRNA Assays (Applied Biosystems, Foster City, CA) and qRT-PCR (data not shown). miR-644a is encoded in intron 14 of E3 ubiquitin-protein ligase itchy homolog (ITCH; NM_031483). Recent reports have shown that several miRNAs, including miR-644a repress androgen receptor expression [4], [5]. Our data identify two target genes of miR-644a – GAPDH and β-actin. It would be interesting to investigate if miR-644a modulates the downstream functions of these two housekeeping genes and consequently, affects glycolysis, cytoskeleton dynamics and cell motility.

### Housekeeping Genes and miRNAs

miRNAs orchestrate posttranscriptional regulation of gene expression mainly by binding to their target sites in the 3′ UTRs of mRNAs and triggering mRNA degradation or translational repression [6]. Most mammalian mRNAs have 3′ UTRs containing target sites for multiple miRNAs. On the other hand, some genes appear to have evolved to avoid miRNA-mediated regulation. Housekeeping genes are considered the classic examples of miRNA non-target genes or “antitargets” [7], [8]. Since housekeeping genes are required for basic cellular processes in all cells, miRNA-mediated repression of their expression would be unfavorable for cell survival. Studies indicate that housekeeping genes circumvent miRNA-mediated regulation by limiting their 3′ UTR lengths [8], [9]. Housekeeping genes appear to have relatively short 3′ UTRs and consequently, they can harbor fewer miRNA target sites. The miRNA target site prediction tools such as TargetScan and PicTar predict multiple miRNA binding sites in the 3′ UTRs of housekeeping genes. However, whether these limited number of target sites and potentially interacting miRNAs play a role in the modulation of housekeeping genes’ expression remains to be determined. A significant biological question is whether the housekeeping gene-targeting miRNAs are expressed ubiquitously in order to maintain stable and constant expression of housekeeping genes in all biological contexts. Alternatively, are these miRNAs expressed at low levels or not expressed at all and consequently, have mild or no effect on the expression of housekeeping genes? Our data demonstrating the repression of GAPDH and β-actin expression by miR-644a suggest that some of the predicted miRNA target sites in the 3′ UTRs of housekeeping genes may indeed be functional. In addition, previous studies have shown the regulation of β-actin expression by miR-145 [10], [11] and miR-206 [12]. Numerous studies have provided evidence for significant variation in the expression of housekeeping genes between different cell types, developmental stages and under different experimental conditions [2], [3], [13], [14]. It is possible that miRNA-mediated regulation is responsible for a portion of this expression variability of housekeeping genes. Interestingly, some housekeeping genes have recently been classified as “disallowed genes” based on their profound repression in specific tissues. The observed disallowance of housekeeping genes has been attributed to epigenetic silencing and miRNA-mediated repression [15].

GAPDH is a key regulatory enzyme, which catalyzes the oxidative phosphorylation of glyceraldehyde-3-phosphate during glycolysis. β-actin is a cytoskeletal protein involved in cell structure and motility. Based on these basic and ubiquitous cellular functions, GAPDH and β-actin are considered as housekeeping genes. However, mounting evidence now recognizes GAPDH and β-actin as multifunctional proteins involved in diverse biological processes independent of their traditional “housekeeping” roles. Several studies have demonstrated the roles of GAPDH in regulation of cytoskeleton [16], membrane fusion and transport [17]–[19], apoptosis [20], [21], DNA repair, DNA replication [22], [23] and regulation of transcription and translation [24]–[27]. In addition, GAPDH has been implicated in the pathophysiology of neurodegenerative diseases [28]. Both GAPDH and β-actin are differentially expressed in several cancers [29]–[33]. β-actin expression has been shown to positively correlate with tumor invasiveness and metastatic potential [34], [35]. Altered expression of β-actin has also been observed in Alzheimer’s disease and Down’s syndrome patients [36], [37]. A recent study reported the translocation of β-actin from cytoplasm to nucleus during macrophage differentiation of HL-60 cells [38]. The nuclear β-actin was found to regulate transcription during macrophage differentiation [38]. Several studies have reported considerable variation in the expression of GAPDH and β-actin between different tissue types and in response to several experimental treatments, demonstrating their differential regulation and hence, their inadequacy to function as reference genes for data normalization [13], [29], [33], [39]–[43]. GAPDH expression has been shown to be modulated by serum, epidermal growth factor, retinoic acid, insulin, norepinephrine, tri-iodothyronine, oestradiol, insulin growth factor 1, basic fibroblast growth factor, 1,25-dihydroxyvitamin D3 and some drugs such as bisphosphonates [39], [43]–[46]. Likewise, some regulators of β-actin include matrigel, hormones, serum, hyperglycemia, hypoxia and tumor necrosis factor-α [33]. Our study adds a new regulator, miR-644a to the growing list of GAPDH and β-actin regulators. In addition to miR-644a, several other miRNAs are predicted to bind GAPDH and β-actin 3′ UTRs and hence, possess the potential to regulate their expression. It may be wise to consider the list of predicted miRNA binding sites in the 3′ UTR of a housekeeping gene before selecting it as an internal control in miRNA experiments. Also, it would be interesting to explore if a subset of miRNAs, which potentially target several housekeeping genes share common characteristics and can be grouped into a separate family.

In conclusion, we advise caution regarding the prevailing assumption of inconsequential effects of miRNAs on the expression of housekeeping genes. Even though miRNAs may not play significant roles in the regulation of housekeeping genes under normal physiological conditions, they may exert measurable effect on housekeeping genes in ectopic overexpression experiments. Hence, in experiments where a miRNA is overexpressed in order to study its effect on target gene expression, careful consideration should be given to the selection of a reference gene.

## Materials and Methods

### Cell Culture

Human prostate cancer cell line, LNCaP was cultured in RPMI 1640 medium supplemented with 10% fetal bovine serum (FBS), 2 mM L-glutamine and antibiotics (100 units/ml of penicillin G sodium; 100 µg/ml of streptomycin sulphate). Human cervical cancer cell line, HeLa and human embryonic kidney derived cell line, HEK 293T were cultured in DMEM supplemented with 10% FBS and antibiotics. Chinese hamster ovary derived cell line, CHO-K1 was cultured in DMEM supplemented with 5% FBS, 2 mM L-glutamine, 1 mM L-proline, 10 mM HEPES and antibiotics. All cell lines were obtained from American Type Culture Collection (Manassas, VA) and maintained in a humidified 5% CO_2_ atmosphere at 37°C.

### Determination of GAPDH, β-actin and STAT2 mRNA Expression by Quantitative Real-time PCR

LNCaP, 293T and HeLa cells were seeded in six-well plates one day prior to transfection. Cells were transfected with miR-644a mimic (50 nM in LNCaP and HeLa; 100 nM in 293T) or negative control (NC) mimic (50 nM) using Lipofectamine 2000 (Invitrogen, Carlsbad, CA). Synthetic miRNA mimics were obtained from Dharmacon (Chicago, IL). Total RNA was isolated from these cells 48 hours post-transfection using Trizol reagent (Invitrogen). 5 µg of total RNA was incubated with 5 units of DNase (Promega, Madison, WI) at 37°C for 40 minutes in order to remove DNA contamination. 1 µg of DNase treated RNA was reverse transcribed into cDNA using the ImProm-II Reverse Transcription System (Promega). Primers for real-time PCR were as follows: (i) GAPDH Forward 5′-ACCCACTCCTCCACCTTTGAC-3′, Reverse 5′-TGTTGCTGTAGCCAAATTCGTT-3′; (ii) β-actin Forward 5′-GCCGGGACCTGACTGACTAC-3′, Reverse 5′- TTCTCCTTAATGTCACGCACGAT-3′; (iii) STAT2 Forward 5′-ACTGAGCCAATGGAAATCTTCAG-3′, Reverse 5′- AAACCTCATCCACGGTGTTCTG-3′; (iv) 18S rRNA Forward 5′-TCGGAACTGAGGCCATGATT-3′, Reverse 5′-CTTTCGCTCTGGTCCGTCTT-3′. The real-time PCR reactions were set up using the SYBR GreenER qPCR SuperMix Universal obtained from Invitrogen. In brief, a 20 µl reaction was set up containing 1X SYBR Green Supermix (Invitrogen), 0.05 µM of each of the forward and reverse primers, 500 nM ROX dye and 1 µl of 1 in 5 diluted template cDNA. For amplification with 18S rRNA primers, 1 µl of 1 in 100 diluted template cDNA was used per reaction. The reactions were dispensed into 96-well optical plates and amplification was carried out in StepOnePlus Real-time PCR System (Applied Biosystems, Foster City, CA) under the following conditions: 50°C for 2 minutes, 95°C for 10 minutes followed by 40 cycles of 95°C for 15 seconds and 60°C for 1 minute. Three replicates were performed per cDNA sample along with the ‘no reverse transcriptase’ and ‘no template’ controls. The specificity of amplification was confirmed by melting curve analysis and also by running PCR products on 3% agarose gels. Gene expression was quantified using the relative standard curve method. Different dilutions of cDNA synthesized from RNA extracted from untreated LNCaP cells were used to plot the standard curves for each gene. GAPDH, β-actin and STAT2 mRNA expression was normalized to 18S rRNA expression. Mean normalized GAPDH, β-actin and STAT2 expression ± SE was calculated from three independent experiments.

### Western Blotting

Proteins were extracted from LNCaP, 293T and HeLa cells transfected with miR-644a mimic (50 nM, 100 nM) or NC mimic (50 nM) 48 hours post-transfection using the M-PER mammalian protein extraction reagent (Pierce, Rockford, IL) containing protease inhibitor and phosphatase inhibitor cocktail (Sigma-Aldrich, St. Louis, MO). The protein concentration in the total cell lysate was determined by Bradford protein assay. 2 µg of protein was resolved on NuPAGE 4–12% Bis-Tris gels (Invitrogen) and electro-transferred to nitrocellulose membranes. Membranes were blocked with 5% nonfat dry milk for 1 hour at room temperature and then incubated overnight with rabbit monoclonal anti-GAPDH antibody (1∶20000, Cell Signaling Technology, Inc., Danvers, MA), mouse monoclonal anti-β-actin antibody (1∶15000, Sigma-Aldrich, St. Louis, MO) and rabbit polyclonal anti-STAT2 antibody (1∶1000, Santa Cruz Biotechnology, Santa Cruz, CA) at 4°C. Blots were washed and incubated with horseradish peroxidase conjugated anti-rabbit (1∶5000, Santa Cruz Biotechnology) and anti-mouse (1∶10000, GE Healthcare, Piscataway, NJ) secondary antibodies for 1 hour at room temperature. At the end of this incubation, blots were washed and treated with ECL Plus Western blotting detection reagent (GE Healthcare). Bands were visualized by exposing to X-ray films. The signal intensities of bands were measured using ImageJ software. The level of GAPDH and β-actin protein expression in each lane was determined by normalizing GAPDH and β-actin band intensity to STAT2 band intensity.

### Luciferase Assays

GAPDH 3′ UTR (200 base pairs; accession number NM_002046.3) and a segment of β-actin 3′ UTR (249 base pairs; accession number NM_001101.3) containing the predicted target site for miR-644a were cloned downstream of firefly luciferase coding region in pMIR-REPORT vector (Ambion, Austin, TX). These constructs were named WT-3′ UTR (WT: wild type). Site-directed mutagenesis of the putative target site for miR-644a in WT-3′ UTR constructs was carried out in order to generate the MUT-3′ UTR constructs using the Change-IT Multiple Mutation Site Directed Mutagenesis kit (USB Corporation, Cleveland, OH). In the GAPDH MUT-3′ UTR construct, 5 nucleotides in the seed matching region of the target site were mutated to their complementary sequence so as to abolish the putative miRNA:target mRNA base-pairing. In the β-actin MUT-3′ UTR construct, 2 nucleotides (AC) in the seed matching region of miR-644a target site were mutated to their complementary sequence (UG). Nucleotide sequences of the constructs were confirmed by DNA sequencing. For luciferase assays, CHO-K1 cells (30,000 cells/well) were plated in 24-well plates one day prior to transfection. Cells were co-transfected using Lipofectamine 2000 reagent (Invitrogen), with 100 ng of WT-3′ UTR or MUT-3′ UTR firefly luciferase reporter construct, 0.5 ng of renilla luciferase reporter plasmid (Promega) and either miR-644a mimic (2 nM) or NC mimic (2 nM). Cell lysates were assayed for firefly and renilla luciferase activities 24 hours after transfection using the Dual-Luciferase Reporter Assay System (Promega) and Victor 3 Multilabel Counter 1420 (PerkinElmer). Renilla luciferase activity served as a control for transfection efficiency. Data are represented as ratio of firefly luciferase activity to renilla luciferase activity. Luciferase assays were repeated at least three times with two replicates each time and substantially similar results were obtained.
